# Kate Field’s Washington

**Published:** 1890-11

**Authors:** 


KATE FIELD'S WASHINGTON is a National Independent Review, published every Wednesday, and mailed to regular subscribers at four dollars a year.

It is a bright, spicy magazine with dashing editorials and interesting miscellaneous articles. It is the famous publication whose author uses the "big I" in her breezy editorials. Accordingly its title-page is decorated with a big I and the spread eagle. For subscriptions apply to KATE FIELD'S WASHINGTON, 59 Corcoran Building, Washington, D. C.

We have received the following:

